# Hospitals of the Women’s Homœopathic Association

**Published:** 1887-07

**Authors:** Walter M. James


### HOSPITALS OF THE WOMEN'S HOMŒOPATHIC ASSOCIATION OF PENNSYLVANIA.

(Twentieth Street and Susquehanna Avenue, Philadelphia.)

In the May number of this journal a plain and simple statement was made in behalf of the lady Managers of the above named hospitals concerning the extraordinary action of eight lady physicians in resigning from the staff of medical attendants. *The Hahnemannian Monthly* in its May number attempts a reply. We have neither the time nor the inclination to make a rejoinder to all the assertions therein given. We will notice only one, which concerns us personally. Quoting from page 320 :

> "One of the ladies alluded in rather forcible terms to a statement published in the May number of THE HOMŒOPATHIC PHYSICIAN, page 179. The writer is one of the signers of the non-interference letter and describes a case of 'uterine cancer in which the putrescent odor was so strong as to sicken the nurse and cause one of the lady Managers to partially faint. He gave the indicated remedy, Silicea 2c with the result of reducing the odor to an extent that surprised the Managers, several of whom were witnesses of this excellent result of applying homœopathic methods'! 'Why,' said the lady, after reading the account, 'the doctor suspended sheets, wet with Platt's Chlorides, in the patient's room, and fairly saturated the apartment with its vapors. *That's* how he reduced the odor and surprised the Managers with Silicea.'"

The lady who thus "alludes in rather forcible terms" to our editorial, in making the above statement is simply guilty of a deliberate perversion of an actual fact, thus identifying herself with the eight seceders who resorted to the same subterfuge when they quoted Dr. Lippe's remark concerning the use of crude drugs. If she wished to effectually fasten upon us the odium of a falsehood, why did she not also mention the lamp generating chlorine gas by burning a mixture of alcohol and chloroform which was brought into the room to "kill" the odor?

Now we did NOT order Platt's Chlorides to be used in that room nor did we recommend the lamp. Both were introduced without our knowledge and consent. We exprsssed our disapproval, but finding on our next visit our advice unheeded, we notified the President, Miss A. E. Ramborger, who at once had the lamp nuisance abated. As for Platt's Chlorides, no farther notice of them was taken, as we were well aware that they could not affect the case. Indeed, they were used excessively for probably forty-eight hours before we took charge of the patient without the slightest effect in reducing the smell.

We are astonished to find that there is any person claiming to be a regularly educated physician who is so ignorant of physics as to suppose that a few square feet of surface wet with

*any* so-called "disinfectant" is competent to allay an odor when the factory—so to speak—for the production of that odor is turning out a fresh supply of it every instant. The author of the "forcible terms" still further betrays her ignorance by talking about the "vapors" from Platt's Chlorides. The inventor of this disinfectant claims that it is absolutely "odorless" and therefore cannot have any "vapor." He publishes its formula, showing it to be a solution of well-known crystallizable salts. Any one having the slightest knowledge of chemistry must know that these salts give off no "vapors" at ordinary temperatures. Yet this woman who thus shows herself incapable of understanding this simple proposition of the legitimate use and limitation of disinfectants, assumes to judge so difficult a subject as the work of the immortal Hahnemann, and to reject portions of it or even the whole of it at her pleasure. A combination of arrogance and ignorance that must invite only laughter and scorn! It is a fair sample of the mode of thought of all these ladies who have resigned.

Since the departure of the malcontents an investigation of the closets and out-of-the-way corners of the Hospital has brought to light vials of Castor Oil, Carbolic Acid, Quinine pills, Ergotine pills, Morphia in 1–16 grain pills, and a bottle of pills compounded of Belladonna, Strychnia, Quinine, and Hyoscyamus. *Perhaps* these doctors call these things homœopathic remedies!

Heaven only knows what harm has been done in this institution with Ergotine, for all eclectic practitioners have just enough knowledge of Homœopathy to repose in crude drugs a blind faith from which any educated allopathist is exempt by reason of his skepticism. Hence these eclectics unwittingly do incalculable damage by pushing such drugs to a degree that the allopathist would not risk.

Plead, defend, extenuate, and misrepresent as they may, these women doctors will not remove the three-fold stigma which they have by their own acts fixed upon themselves. *Firstly*, by pretending to be homœopathists and then practicing what is *not* Homœopathy. *Secondly*, proving so recreant to the trust reposed in them at the Hospital, that they slyly introduce their corrupt medical methods into its wards in defiance of the rules, and continue in such practice until they are discovered and checked by the laity for this departure from their own professions. *Thirdly*, when remonstrated with, they seek revenge and display their want of self-respect by resorting to the infamous "boycott," and that, too, against an institution for *charity!*

WALTER M. JAMES.

